# Supplementary material for: Learning spatio-temporal patterns with Neural Cellular Automata
Source: PLoS Comput Biol. 2024 Apr 26;20(4):e1011589. doi: 10.1371/journal.pcbi.1011589 (PMC11078362; doi:10.1371/journal.pcbi.1011589)
Supplement: S1 Appendix — (PDF) [file pcbi.1011589.s001.pdf]

Learning spatio-temporal patterns with Neural Cellular Automata

Supporting information

Alex D. Richardson[1,2,*], Tibor Antal [2], Richard A. Blythe [1], Linus J. Schumacher [2,3],

* alexander.richardson@gmail.com
1 School of Physics and Astronomy, University of Edinburgh, Edinburgh, UK
2 School of Mathematics and Maxwell Institute for Mathematical Sciences, University of Edinburgh, Edinburgh, UK
3 Institute of regeneration and repair, University of Edinburgh, Edinburgh, UK

1 Gradient Calculation

To compute the gradients, we use the shorthand $\varphi_j = x^{((m-1)t+j)}$, that is the NCA state propagated from time $(m-1)t$ by j time steps, which we can write step by step as

$$\begin{aligned}
\varphi_0 &= x^{((m-1)t)} \\
\varphi_1 &= \Phi(\varphi_0, \theta) = x^{((m-1)t+1)} \\
&\vdots \\
\varphi_j &= \Phi(\varphi_{j-1}, \theta) = \Phi^j(\varphi_0, \theta) \\
&\vdots \\
\varphi_t &= \Phi(\varphi_{t-1}, \theta) = \Phi^t(\varphi_0, \theta) = x^{(mt)}
\end{aligned}$$

We have a distance $\mathcal{L}(x, y)$ between two states. We want to minimize the total distance between the NCA prediction and the data averaged over all m in terms of the parameters θ, so we look for

$$\frac{d}{d\theta}\frac{1}{D}\sum_m \mathcal{L}(x^{(mt)}, y^{(m)}) = \frac{1}{D}\sum_m \frac{\partial \mathcal{L}}{\partial x^{(mt)}}\frac{dx^{(mt)}}{d\theta}$$

since only the prediction $x^{(mt)}$ depends on the parameters θ and not the distance function or the data. The second term can be written as

$$\frac{dx^{(mt)}}{d\theta} = \frac{d\varphi_t}{d\theta} = \frac{d\Phi(\varphi_{t-1}, \theta)}{d\theta} = \frac{\partial \Phi(\varphi_{t-1}, \theta)}{\partial \varphi_{t-1}}\frac{d\varphi_{t-1}}{d\theta} + \frac{\partial \Phi(\varphi_{t-1}, \theta)}{\partial \theta}$$

We decreased the subscript t, which we can do in general

$$\frac{d\varphi_j}{d\theta} = \frac{d\Phi(\varphi_{j-1}, \theta)}{d\theta} = \frac{\partial \Phi(\varphi_{j-1}, \theta)}{\partial \varphi_{j-1}}\frac{d\varphi_{j-1}}{d\theta} + \frac{\partial \Phi(\varphi_{j-1}, \theta)}{\partial \theta}$$

all the way down to φ_0. We get that

$$\begin{aligned}
\frac{dx^{(mt)}}{d\theta} = \frac{d\varphi_t}{d\theta} = \frac{\partial \Phi(\varphi_{t-1}, \theta)}{\partial \varphi_{t-1}}\left(\frac{\partial \Phi(\varphi_{t-2}, \theta)}{\partial \varphi_{t-2}} \cdots \left(\frac{\partial \Phi(\varphi_0, \theta)}{\partial \varphi_0}\frac{d\varphi_0}{d\theta} + \frac{\partial \Phi(\varphi_0, \theta)}{\partial \theta}\right)\right. \\
\left. + \cdots + \frac{\partial \Phi(\varphi_{t-2}, \theta)}{\partial \theta}\right) + \frac{\partial \Phi(\varphi_{t-1}, \theta)}{\partial \theta}
\end{aligned}$$

Here it is important to note that each partial gradient $\frac{\partial \Phi(\varphi_i, \theta)}{\partial \varphi_i}$ and $\frac{\partial \Phi(\varphi_i, \theta)}{\partial \theta}$ are matrices of size $|\mathcal{X}| \times |\mathcal{X}|$ and $|\theta| \times |\mathcal{X}|$ respectively. Although in practice there will be nice factoring to simplify the computation, the memory requirement is still dominated by $|\mathcal{X}|^2$. This computation is hidden in line 17 of Algorithm 2 for brevity.
