# Supplementary material for: Learning spatio-temporal patterns with Neural Cellular Automata
Source: PLoS Comput Biol. 2024 Apr 26;20(4):e1011589. doi: 10.1371/journal.pcbi.1011589 (PMC11078362; doi:10.1371/journal.pcbi.1011589)
Supplement: Attachment — Submitted filename: reviewer_response.pdf [file pcbi.1011589.s008.pdf]

1 Reviewer 1

> The paper presents an interesting approach to modelling emergent spatio-temporal patterns using Neural Cellular Automata (NCA). The authors show that NCA can learn dynamics from snapshots of patterns at multiple timepoints and can infer update rules equivalent to those obtained by discretizing and iterating partial differential equations (PDEs) commonly used in biological pattern formation.
>
> This is an excellent paper and I am excited about the future work that it can lead to.
>
> The only errors I can find are typographical. At the end of page 12, where "it's" should be "it" and in the caption of fig 8 C and D are separated by a period, rather than a comma.

We thank the reviewer for their positive assessment, and have corrected these typographical errors.

> I am also very grateful for the authors releasing the code and I hope people take it up.
>
> The rest of my comments are either questions, or requests, for further clarifications.
>
> 1) During the simulations (fig 3, for example) generating learning data the final time step does not appear to be a steady state. Does this influence the learning of the NCA?

The reviewer has correctly identified that in our training data the PDEs do not reach steady state, which we had not made sufficiently clear. We are specifically interested in learning the transient dynamics, as might be more relevant in biological applications, and which is arguably a harder problem. We now clarify this in our revised manuscript in section 3.1.

> 2) Figure 4b confuses me. The times are mixed with PDE results and are in a grid rather than given as a line, as in fig 5, which is much clearer.

We agree that Figure 4b was easy to misread in that regard, and have restructured Figure 4b, and used a similar structure for the new figure (now figure 6) we added.

> 3) Could the authors add a paragraph to the discussion regarding the potential of their work? Namely, I assume the overall future aim of this work would be to make the rules that the NCA learn to be interpretable; can we understand what the NCAs are doing, rather than using a blackbox neural network? Moreover, how unique are the rules that the NCA learn? The links in with the recent paper Woolley, T.E., Krause, A.L. & Gaffney, E.A. Bespoke Turing Systems. Bull Math Biol 83, 41 (2021). `https://doi.org/10.1007/s11538-021-00870-y` which shows that Turing systems are generally underconstrained so there are infinite families of systems that will produce similar patterns.

The interpretability of the NCA is indeed a good point, the reviewer is pre-empting some natural next steps of research. We have added to the discussion on interpretability ("The minimalism of the NCA model...", bottom of page 27, and "It is also worth stating that interpretability...", page 28), specifically highlighting areas we think are a good place to start with further research. The point about non-uniqueness is subtle but important, so we have elaborated on this in the discussion ("It is worth emphasising the non-uniqueness...", page 25), citing the paper: Woolley, T.E., Krause, A.L. & Gaffney, E.A. Bespoke Turing Systems. Bull Math Biol 83, 41 (2021).

2 Reviewer 2

> In this paper, the authors present an advancement in the field of Neural Cellular Automata (NCA), with the aim of bridging machine learning and mechanistic modelling. To do so, the authors have trained NCA to decipher complex dynamics from image time series and PDE trajectories, focusing on uncovering local rules that dictate large-scale dynamic and emergent behaviors. Notably, they expand the application of NCA beyond the conventional realm of learning stationary emergent structures. The paper demonstrates the generalization capabilities of NCA beyond PDE training data, and it offers insightful exploration into constraining NCA to adhere to specific symmetries, and the impacts of hyperparameters. I particularly appreciated the clear explanation of the atrchitecture of the network and its training process, a detail often omitted in AI literature. This clarity makes the paper accessible to a wide audience. However, the paper's primary limitation is its limited alignment with computational biology, especially in the image morphing section. Additional comments are provided below.

We thank the reviewer for their positive comments, especially their appreciation of our detailed explanation of architecture and training process.

2.1 Major comments

> 1) As explained in the introduction, one of the motivations of learning the rules of cellular automatas is solving the inverse problem of learning the rules behind a biophysical process. This is pursued further in the network architecture trying to generate a network that can be interpreted easily. Nevertheless, the authors did not analyze the resulting trained network e.g. what can recover/learn about the PDE once the network is trained? I can't think of any biological question in which I would care about the future dynamics of an image without being interested in extracting the mechanistic process underneath. Questions that would be interesting are:
>
> 1a) Can the diffusion rates be inferred from the training?

1b) Does the structure of the hidden layers give clues on the first and third order reactions?

1c) Are their parameter sets for which the training fails or is less informative?

We agree that interpreting and analysing the trained network is desirable, although believe that to do so properly would make this paper too long. The full analysis and interpretation deserves its own paper, especially if tied to a specific biological application. We are currently working on such a follow-up paper. We have however added a sizeable text in the discussion ("The minimalism of the NCA model...", bottom of page 27, and "It is also worth stating that interpretability...", page 28), giving some guidance and detail on what we believe to be the most promising directions for interpretation and analysis.

2) As explained in the second part of the manuscript, it is interesting to understand predictions under noise perturbations. This is also true for the PDE problem since experimental data will always contain some noise. How robust is the PDE training to noisy data? My guess is that it should work, but it would be good to see it in action as in the image morphing example.

The reviewer has enquired about how noise affects NCA on the PDE task. Given that we explore noise on the Image Morphing task, it is a natural addition to include some results about how noise affects the training process of NCAs on the PDE task. Following up on the reviewer's advise, we find that NCA training is fairly robust to noise, but the failure cases are also illuminating in how the NCA are biased towards learning patterning. We have included this in Section 3.1.2 in the revised manuscript, and have added a new Figure 6 (renumbering subsequent ones).

3) There are some comments in the discussion about the comparison of NCA with other similar AI approaches for PDE inference such as SINDy, but I found they were a bit limited. Since this paper shows a method that connects AI learning to PDE inference, I would like to know more about how these approaches compare. What are the limitations of both? What problems would be more suitable for one or the other? Are there any difference in the training constrains?

We agree that a clearer comparison between our method and others is valuable, so we have elaborated further ("NCA have strong performance on...", top of page 27). In short, SINDy and NCA are very different in their technical details, but have some overlap in the kinds of problems that are trying to be modelled. The main technical difference is SINDy requires more data, and requires time derivatives of that data, whereas NCA are capable with sparse data.

4) I did not understand the biological relevance of the image morphing study. I appreciate that the authors chose a simplistic emoji problem to make the analysis clear and general. Nevertheless, a significant text is missing supporting the motivation of this problem in a computational biology context.

We appreciate the reviewer's understanding that the emoji problem was a tractable toy problem to facilitate development of techniques. We have however elaborated in the discussion ("Of particular biological interest...", bottom of page 25) on the sorts of biological systems that we believe NCA are suited to.

2.2 Minor comments

> 1) Along the paper, Sobel kernels are used as a synonym of gradient. But Sobel kernels also generate some smoothing of the data. This is not clear when they are connected to PDE operators or biophysical processes. I don't think this is a problem in the training, as the gradient operator can probably be expressed as a combination of the 5 kernels used. This should be clarified.

We agree that the interchanging of nomenclature here is unnecessary and potentially confusing, so we have cleaned this up throughout the manuscript. We refer to the kernels as gradients throughout, in line with our calling the Laplacian kernel the Laplacian (rather than a specific discretisation). When introducing the kernels in section 2.1, we state that we use Sobel filters as gradient approximations. There are other discrete approximations of gradients, but we don't believe this would make much difference. The initial papers that introduce NCA also use Sobel filters as gradient approximations, so we choose to remain consistent with this literature.

> 2) The description of the training was very clear, and I really appreciate the effort the authors put into writing this section. There is a minor abuse of notation in the initialisation of the variables and comparing the loss - "We initialise $x(\delta t) = y(\delta)$" vs "we compare $x(\delta t)$ to $y(\delta)$"). Perhaps it would be good to indicate the initial points with a hat as it is done in Algorithm 2?

We thank the reviewer for spotting this inconsistency of notation. We have changed the text in section 2.2 to use $\hat{x}$ instead of x where appropriate, consistent with algorithm 2. We hope this makes this necessarily dense section easier to understand.

> 3) I also found a bit confusing the δ notation e.g. $y(\delta)$ vs $x(\delta t)$. I can't think of a better candidate right now, but for me it broke a bit the reading flow of the text, for instance when interpreting Figure 2.

We agree that the original notation was slightly poorly-chosen, particularly since δt could be misread as a time interval, rather than the product of an integer with a time integral. We have chosen different letters to avoid this potential misreading, and added some text to further clarify the notation.

> 4) Figs. 4 and 5 could be clearer. On one hand 4B could reorganize the panels to make clear the order/hierarchy of the images. It would also be good to have some spatial difference (or loss) between the PDE and the different NCA solutions, so it is more evident which parts of the solution are not well recovered. Similar thing for Figure 5.

We agree, and this is also brought up by reviewer 1. We have simplified the layout of Figure 4b by splitting into a 4b and 4c. We have also used this improved layout for the new Figure 6, which we added to show how noise affects training in the PDE case. We feel that adding spatial loss to figure 4 would make it overcrowded. We have however added spatial loss to figure 5, as it fits nicely.

> 5) In 3.1 "DA and DB re-scale the patterns, and must be chosen in line with the timestep size to achieve numerical stability". Should not the timestep be chosen according to diffusion rate, not the other way around?

We appreciate the reviewer's attention to detail here, however both the diffusion rates and timestep need to be tuned for stability (indeed their ratio is what matters). For simplicity we set the timestep to 1, and then choose the diffusion rates to both: ensure stability; and scale the spatial resolution of the observed patterning. We certainly could fix the diffusion constants and then tune the timestep, although it would make little difference in this context.

> 6) Boundary conditions for the PDE are periodic, but this is not mentioned until the image morphing section.

We thank the reviewer for spotting this omission. We have added a sentence in section 3.1 stating that the PDE boundary conditions are periodic.

> 7) This comment is related to one of my major comments. In section 3.2.1 "removing global synchronisation between each cell acts like dropout regularisation, and can be biologically motivate". I failed to understand this comment, since there was no reference to biology prior to this comment in the image morphing scenario.

The comment on this being 'biologically motivated' has been removed - it doesn't add much, and is a bit out of place.

3 Reviewer 3

3.1 Overview

> The manuscript by Richardson et al. explores the extension of cellular automata approaches, integrating neural network architectures to learn local rules from dynamic and transient states, as opposed to static images. This novel approach is demonstrated through two applications: learning Turing patterns from PDE outputs and transitioning between images using emojis. The methodology, grounded in a minimalist design with a single hidden layer, is both innovative and intriguing. The paper also examines the stability of these models against variations in initial conditions. While the work is technically robust and presents an interesting concept, there are aspects that could be improved to align more closely with the scope and expectations of PLoS Computational Biology.

We thank the reviewer for their positive review, and their appreciation of minimalist machine learning models.

3.2 Comments and Suggestions

1. Technical Focus and Readability

   The manuscript is heavily technical, with even the Results section focusing primarily on methods. While the methods are thoroughly and clearly explained, this technical orientation may be more suitable for a specialized journal. Consider revising to include more application-focused results or discussions to broaden the appeal for a PLoS CB audience.

   Suggestion: Enhance Fig. 1 by incorporating numbered steps in the schematic and a corresponding detailed explanation. This would aid in making the figure more accessible and self-explanatory to readers who may not be deeply familiar with the technical aspects.

We appreciate the reviewer's concern, however we feel that NCA provide a promising modelling framework that we would like computational biology researchers to be aware of. We have added discussion on future applications to biological systems ("Of particular biological interest...", bottom of page 25). We have rearranged figure 1 slightly, adding alphabetical labels to each section and corresponding paragraphs in section 2.1. We hope this makes the figure more accessible.

2. Biological Relevance

   The connection to biological systems and processes is not strongly articulated (emojis are not sufficient). PLoS CB emphasizes biological insights, and this paper could benefit from a clearer exposition on this front.

   Suggestion: Expand the discussion on how the local rules derived from the model correlate with biological phenomena, such as self-repair or regeneration. Are there implications or insights that this model provides in understanding these biological processes?

We appreciate the need for stronger links to biological insights. As suggested, we have elaborated in the discussion ("of particular biological interest...", bottom of page 25) on potential application on regenerative / self repairing systems. In particular, some of the original literature on NCA explores regenerative behaviour, but only on toy problems. We point out that (to our knowledge), the full potential of modelling biologically realistic regeneration has not been realised, but we believe our work on learning dynamics takes NCA a step closer to modelling such systems.

3. Explanation of PDE Correspondence

The paper mentions that the rules derived correspond to discretized solutions of PDEs, but this aspect is not elaborated sufficiently.

Suggestion: Provide a more detailed explanation or a dedicated section on how these rules relate to the discretized solutions of PDEs, and whether this draws parallels with Turing's original models of discrete cellular systems using hopping instead of diffusion.

We have added a short paragraph near the end of section 2.1 where we derive the functional form of the NCA from a PDE discretisation. This helps to highlight the wide range of potential systems NCA can model.

4. Parameter Sensitivity Analysis

An investigation into the sensitivity of the model to its parameters is absent. Such an analysis could be crucial, especially in the context of robustness and biological relevance.

Suggestion: Conduct a sensitivity analysis to understand how variations in parameters affect the model's output. This would not only enhance the robustness of the model but also provide insights into the robustness problem in Turing systems.

We appreciate the importance of parameter sensitivity analysis, but we feel that to do this properly would make the paper too long. This would naturally fit in with model interpretation, so for now we offer some comments on this in the discussion (top of page 28). We agree that it is worth doing, but would like to fully address this in a follow-up work.

3.3 Conclusion

The paper by Richardson presents a novel and technically sound approach to learning spatio-temporal patterns using neural cellular automata. However, to align more closely with the readership and scope of PLoS Computational Biology, it would benefit from significant enhancements in readability, biological relevance, detailed explanations of key concepts, and robustness analysis. These improvements could significantly elevate the impact and applicability of the research within the field.
